# Early Development of Neural Speech Encoding Depends on Age but Not Native Language Status: Evidence From Lexical Tone

**DOI:** 10.1162/nol_a_00049

**Published:** 2022-02-10

**Authors:** Nikolay Novitskiy, Akshay R. Maggu, Ching Man Lai, Peggy H. Y. Chan, Kay H. Y. Wong, Hugh Simon Lam, Tak Yeung Leung, Ting Fan Leung, Patrick C. M. Wong

**Affiliations:** Department of Linguistics and Modern Languages, Brain and Mind Institute, The Chinese University of Hong Kong, Hong Kong SAR, China; O-lab, Duke Psychology and Neuroscience, Duke University, Durham, NC, USA; Department of Paediatrics, The Chinese University of Hong Kong, Hong Kong SAR, China; Department of Obsterics and Gynaecology, The Chinese University of Hong Kong, Hong Kong SAR, China

**Keywords:** infants, tone language, electroencephalography, frequency-following response, perceptual narrowing

## Abstract

We investigated the development of early-latency and long-latency brain responses to native and non-native speech to shed light on the neurophysiological underpinnings of perceptual narrowing and early language development. Specifically, we postulated a two-level process to explain the decrease in sensitivity to non-native phonemes toward the end of infancy. Neurons at the earlier stages of the ascending auditory pathway mature rapidly during infancy facilitating the encoding of both native and non-native sounds. This growth enables neurons at the later stages of the auditory pathway to assign phonological status to speech according to the infant’s native language environment. To test this hypothesis, we collected early-latency and long-latency neural responses to native and non-native lexical tones from 85 Cantonese-learning children aged between 23 days and 24 months, 16 days. As expected, a broad range of presumably subcortical early-latency neural encoding measures grew rapidly and substantially during the first two years for both native and non-native tones. By contrast, long-latency cortical electrophysiological changes occurred on a much slower scale and showed sensitivity to nativeness at around six months. Our study provided a comprehensive understanding of early language development by revealing the complementary roles of earlier and later stages of speech processing in the developing brain.

## INTRODUCTION

During early infancy, infants with normal hearing are able to perceive a large inventory of speech sounds that are both within and outside of their native language environments. Toward the end of infancy, this broad perceptual ability is gradually reduced to a smaller inventory of sounds that are mostly confined to the infants’ native language environments (Werker & Tees, 1984). The term *perceptual narrowing* or *attunement* has been used to describe this phenomenon, and it is generally viewed as the basis of early language acquisition (Kuhl, 2014; Werker et al., 2012). This perceptual narrowing has a neurophysiological basis. Kuhl postulated that exposure to a particular language results in the development of dedicated (committed) neural networks which code the patterns of the language in question, and also make the learning of other languages more difficult (Kuhl, 2004). This native language neural commitment (NLNC) hypothesis has been influential in shaping our current understanding of how the nervous system supports perceptual narrowing and early language acquisition.

While the NLNC is able to explain a variety of behavioral and neurophysiological (in particular, cortical) data, its current rendition does not account for two important facts about speech sound processing in the developing nervous system. First, although infants in the first six months of life are able to perceive a large inventory of sounds, they are unable to perceive *all* sounds. In fact, it seems to be the case that infants, regardless of their language environment, have difficulty in discriminating speech sounds that are less acoustically salient (Best et al., 1995, 2016). In order for infants to distinguish whether or not a speech sound belongs to its native language and to use it contrastively in lexical contexts, they must first encode and recognize it. Second, speech sound development (and language acquisition) depends on a nervous system that is also developing, regardless of the specific language input, and this helps to determine how well infants can encode speech. Therefore, a comprehensive theory about early language acquisition must be situated in the context of neural development as well as language experience. The aim of the present study is to examine the development of speech perception in the context of both cortical and subcortical neural development during the first two years of life. Our main question is whether perceptual attunement can be explained by developmental changes in neural functions.

The auditory neural system that supports hearing and spoken language consists of both subcortical (including the auditory brain stem) and cortical structures that develop at different rates. In terms of anatomical growth, the connections in the auditory brain stem acquire their mature pattern by the 29th week of gestation and their myelination is completed within the first postnatal year (Hartley et al., 2010; Moore & Linthicum, 2001, 2007; Moore et al., 1995, 1997; Romand & Romand, 1982; Romand et al., 1976; Sano et al., 2007). The 6th postnatal month sees the maturation of the brain stem dendritic trees (Moore et al., 1998). The subcortical-cortical connections, on the other hand, only mature when children are between 3 and 5 years old, and the myelination of cortical layers continues up to the age of 11 or 12 (Moore & Linthicum, 2007).

In terms of functional growth, research on the human auditory neural systems has traditionally relied on scalp-recorded electroencephalography (EEG). Cortical and subcortical EEG components can traditionally be separated by their timing and frequency composition. High-frequency *early-latency* frequency-following response (FFR) is phase-locked to the spectral envelope of an auditory stimulus (Moushegian et al., 1973). Although much more research is needed to understand the sources of the FFR at different frequency ranges (Coffey et al., 2019), the available evidence has suggested that the FFR has stronger (though not exclusive) subcortical generators, including the inferior colliculi, especially for frequencies above 150 Hz (Bidelman, 2018; Tichko & Skoe, 2017). The field seems to be more certain that the auditory *long-latency* response (LLR) is generated exclusively in the cortex, within superior temporal gyri in the vicinity of the Heschl’s gyrus (Godey et al., 2001) where intracranial recordings have shown the presence of phoneme categorical representations (Chang et al., 2010; Mesgarani et al., 2014). The adult LLRs in the time range between 50 and 240 ms are sensitive to the nativeness of phonemes in both active discrimination and passive listening (Gansonre et al., 2018; Kuuluvainen et al., 2014; Näätänen et al., 1997; Ross & Tremblay, 2009; Shtyrov et al., 2000; Tremblay & Kraus, 2002; Tremblay et al., 1998, 2001, 2014).

The FFRs to speech are clearly detectable at birth (Ribas-Prats et al., 2019). The time and frequency-domain parameters of FFR to consonants and vowels improve steadily from birth to 11 years (Anderson et al., 2012, 2015; Skoe et al., 2015). Neonates display a much less matured FFR to lexical tones, pitch patterns that are used to contrast word meaning, than do adults (Jeng et al., 2011, 2016). Although not much is known about the development of FFR to lexical tones in the first few years of life beyond the neonatal period, quite a few research studies have examined FFR to speech more generally in adulthood. Lifelong experience of lexical tones results in enhanced FFR encoding to lexical tones in tone language speakers relative to non-tone language speakers (Krishnan et al., 2005, 2009). Experience with other tone languages, e.g., Thai, may benefit the neural representation of lexical tones of another tone language (e.g., Mandarin; Krishnan et al., 2010). With short-term training, FFR to lexical tones can be improved in native speakers of a non-tone language (Reetzke et al., 2018; Song et al., 2008). Experience-dependent FFR responses may be partially mediated by cortico-fugal projections to the auditory brain stem (Intartaglia et al., 2016; Krishnan et al., 2005; Suga, 2008; Suga et al., 2000; Zhao & Kuhl, 2018) but may also manifest an online adaptation to ongoing statistics in the brain stem (Alho et al., 2019; Escera, 2017; Slabu et al., 2012). These results clearly demonstrate that the FFRs may be a useful tool for studying the neural development of speech. As far as we are aware, studies have yet to investigate the early development of FFR to native and non-native sounds during the period of rapid speech development.

Relative to FFR, a larger body of research has examined the LLR in relation to early speech development. LLR at term birth is dominated by a wide and late positive deflection peaking in the 200–300 ms interval (Barnet & Lodge, 1967; Barnet et al., 1975; Kushnerenko et al., 2002; Novak et al., 1989; Wunderlich & Cone-Wesson, 2006). Later in life the original positivity splits into P1 and P2, the trough between the positive peaks deepens and eventually by 12–16 years old, it becomes the N1 wave of the adult auditory event-related potential (ERP) (Cone & Whitaker, 2013; Lippé et al., 2009; Novak et al., 1989; Ponton et al., 2002; Shafer et al., 2015; Small et al., 2018). (Throughout this article, we use the term *P1* for the largest infant positivity and consider it the homologue of the whole P1-N1-P2 complex in the adult LLRs). More specific to speech, Kuhl and colleagues (Kuhl, 2010) conducted a series of studies to examine the cortical development of native and non-native speech perception. They employed mismatch negativity (MMN), a pre-attentive component of the LLR, that serves as an electrophysiological index of sound discrimination (Cheour-Luhtanen et al., 1995; Partanen et al., 2013). The MMN to various vowel contrasts is present in newborns (Cheour-Luhtanen et al., 1995), but it reduces in response to non-native vowel contrast after 6 months of age (Cheour et al., 1998; Ortiz-Mantilla et al., 2013). Neural commitment to the native language at the age of 7.5 months can predict language development up to two years following initial neural measurements (Kuhl et al., 2008; Rivera-Gaxiola, Klarman, et al., 2005; Rivera-Gaxiola, Silva-Pereyra, & Kuhl, 2005). A recent study of acoustic change complex in the infant LLR demonstrated a transformation from a merely acoustical-driven pattern to native phoneme-category sensitivity around the age of 6 months (McCarthy et al., 2019). Though the findings from these pioneering LLR-based (mostly MMN) studies provide an initial neural explanation for perceptual narrowing, these studies are focused largely on the role of the cortex. In order to obtain a more comprehensive understanding of the development of speech perception in the context of neural development, further research is needed that examines both cortical and subcortical levels of the auditory nervous system.

### The Current Study

In the present study, we examined the functional development of neural responses with generators in the brain stem and cortex in relation to native and non-native speech sounds. As the anatomical and neurophysiological development of the brain stem generally occurs earlier than that of the cortex, we postulate that the function of the brain stem in the first years of life is solely to increase the encoding accuracy of the acoustic input, regardless of its nativeness status. Thus, we predict a beneficial effect of age on the early-latency FFR with no interaction between age and nativeness status during the first two years of life. An increase in encoding fidelity would then allow the cortex to differentiate between native and non-native sounds, enabling greater neural resources to be devoted to native sounds to facilitate infants’ phonological and lexical development. By contrast, long-latency cortical LLR to speech develop more slowly, and the responses show sensitivity to the nativeness status of the speech stimulus. We expect that the already known cortical sensitivity to the nativeness status of a language extends also to the P1 component of the infant LLR as they reflect interacting cortical processes.

**Figure 1.**
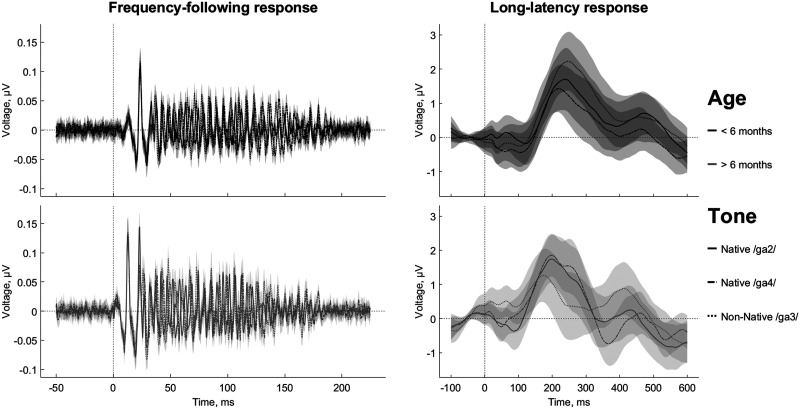
The waveforms of the early-latency and long-latency responses differ in their timing, temporal structures, and maturational patterns. The *left panel* shows the frequency-following response generated predominantly in the brain stem. The *right panel* shows the waveforms of the long-latency responses generated in the cortex. Note the difference in time and amplitude scales between the two panels. The rows correspond to the age groups younger and older than 6 months that are also coded with darker and lighter shades of grey correspondingly. The three lines represent each of the three lexical tones: Native /ga2/ (solid), Native /ga4/ (dash-dotted), and Non-Native /ga3/ (dotted).

Early-latency neural encoding FFR and long-latency cortical LLR recordings were simultaneously collected via EEG (Figure 1) from 85 Cantonese-learning children as they heard two native (Cantonese) and one non-native (Mandarin) speech stimuli that were different only in their pitch (Supplementary Figure 1; Supporting Information (SI) can be found at https://doi.org/10.1162/nol_a_00049). Both Cantonese and Mandarin are tone languages. Lexical tones are ideal for testing our hypothesis because phase-locking to fundamental frequencies that represent lexical tones can be measured by FFR. Children’s EEG responses were collected longitudinally at up to 4 time points (Supplementary Figure 2). Since not every child had a recording at every time point, the final data set represented a mixture of longitudinal trajectories and cross-sectional data points. We employed mixed-effects models that took account of repeated measures when the data were available within the same subjects. This design is similar to other studies of neural development (Raznahan et al., 2014; Wierenga et al., 2014).

We expected measures of FFR to improve linearly and substantially across age during the early stages of development (23 days to 24; 16 months), as predicted by the literature on brainstem development (Moore et al., 1998). Importantly, improvement in these FFR measures can be observed in both native and non-native tones. By contrast, LLR measures are likely to improve more slowly than those of early-latency neural encoding measures within the same developmental period because the auditory cortex takes longer to develop (Moore & Linthicum, 2007). As in previous studies of speech development in the cortex, we expected to see sensitivity to the nativeness status of speech sounds occurring at about 6 months (Ortiz-Mantilla et al., 2013), because 6 months is when cortical responses to lexical tones are found to be adultlike (Cheng et al., 2013). As lexical tone is a type of speech sound that occurs in every syllable in a tone language and about 70% of the languages in the world are tone languages (Fromkin, 2014; Yip, 2002), we believe an investigation into the cortical-subcortical development of speech using lexical tone as a starting point is a reasonable one. Our results can lay the foundation for future research that examines other categories of speech sounds.

## MATERIALS AND METHODS

### Participants

Eighty-five Cantonese-learning infants and toddlers (48 males) between 23 days and 24; 16 months of age (mean age 7; 5 months, *SD* 5; 9 months), with no significant medical history, participated in this study. Most of the participants were recorded longitudinally with up to 4 visits 5; 7 months ± 7.2 days apart on an average, producing 177 data points (Supplementary Figure 2). By self report, parents were native Cantonese speakers and spoke Cantonese at home. Note that although English and Mandarin (Putonghua) are languages spoken by residents of and visitors to Hong Kong and are taught in the public schools, the vast majority of the population speaks Cantonese (88.9%) as their usual language, with English (4.3%) and Mandarin (1.9%) being the clear minorities (Government of Hong Kong SAR, 2016).

Written informed consent was obtained from parents or guardians prior to their child’s participation in the study. The experimental protocol was in compliance with the Declaration of Helsinki.

### Stimuli and Experimental Design

The lexical tones were Cantonese (Native) tones 2 and 4 (rising and falling) and Mandarin (Non-Native) tone 3 (dipping) embedded in the syllable /ga/. We chose the speech stimuli based on two principles: (1) The native tone categories must be distinctive; tone 2 and tone 4 being of different contour shapes fulfilled this requirement. (2) The native and non-native tone categories must also be distinctive. Mandarin tone 3 is quite unique and does not overlap with any Cantonese tones. Furthermore, these same speech stimuli were used in our previous study (Maggu et al., 2018), attesting to their validity for obtaining robust FFR responses.

The stimuli were spoken by a phonetically-trained female speaker, and were duration and amplitude normalized. The fundamental frequency (F0) ranges for the Native /ga2/, Native /ga4/, and Non-Native /ga3/ were respectively 182–278 Hz, 152–187 Hz, and 142–177 Hz (Supplementary Figure 1). These three tones are described as 25, 21, and 214, respectively, according to Chao’s nomenclature (Chao, 1930). Those frequencies are well above the frequency range for cortical generators to be the dominant sources (Bidelman et al., 2018). Non-native tone was acoustically more complex than the other tones (see SI). This acoustic complexity was not a confound because our results suggested that the Native /ga2/ tone posed the most difficulty for the listeners (see SI). The stimuli were presented to the participants via Audio CPT module of STIM2 (Compumedics, Australia). One thousand repetitions of the tones were delivered in separate blocks to both ears of the participant with fixed interstimulus interval 500 ms (stimulus-onset-asynchrony 674 ms) and alternating polarity.

### EEG Recording and Analysis

Continuous EEG was collected using a Synamps RT amplifier connected to a Curry 7.05 workstation (Compumedics, Australia) from five Ag/AgCl electrodes at Cz, M1 (left mastoid) and M2 (right mastoid) at a sampling rate of 20 kHz with CPz as reference and Fpz as ground. Interelectrode impedances were maintained at less than or equal to 1 kΩ. Cz data were re-referenced offline with the average of two mastoids. The subsequent analysis was performed on Cz. All data analysis was performed in MATLAB 9.2 (R2017a; mathworks.com), with custom-made scripts using built-in functions of EEGlab (Delorme & Makeig, 2004), ERPLAB (Lopez-Calderon & Luck, 2014), Signal Processing Toolbox (mathworks.com), and Statistics and Machine Learning Toolbox (mathworks.com). Two different analysis pipelines were used for FFR and LLR extraction correspondingly (see below). For each of the early-latency neural encoding FFR and long-latency cortical LLR responses, we examined a number of conventional metrics to investigate their developmental trajectories. An important consideration was whether the native and non-native tones differ in their developmental trajectories of the auditory early-latency neural encoding and the long-latency cortical responses.

Our recording procedures allowed us to simultaneously collect early-latency FFR and long-latency LLR with the same set of stimuli in the same child. As far as we are aware, this is the first study to simultaneously record FFR and LLR in infants; only adult studies of the kind have been performed to date (Alho et al., 2019; Shiga et al., 2015). For each of the early-latency FFR and long-latency cortical LLR measures, we examined a number of conventional metrics (Anderson et al., 2012, 2015; Jeng et al., 2011, 2013, 2016; Skoe et al., 2015) to investigate their developmental trajectories. The aim was to trace all the subjects longitudinally. However, the main challenge of a longitudinal design was subject attrition, which turned this study into a mixed cross-sectional and longitudinal design. Additionally, the EEG recording in infants is prone to movement artifacts and not every recording session is successful. One option would be keeping only the subjects with a certain amount of longitudinal data points. The size of our sample would allow us such a manipulation. However that would not only reduce statistical power but also render the sample unrepresentative in relation to the original population. We opted to keep all subjects who had at least one successfully recorded EEG session. To take advantage of the fact that repeated measurements were taken from some participants, we employed mixed-effects models with subject as a factor. Our primary focus was to understand whether the growth of FFR measures was rapid, and whether this growth could be observed in both native and non-native tones. Our secondary focus was to understand whether the growth of the LLR measures appeared to be less substantial, and whether sensitivity to nativeness could be seen at around 6 months of age.

### FFR Analysis

For FFR extraction, the data were filtered with an 8-order Butterworth 80–1500 Hz bandpass filter and down-sampled to 3 kHz. The epochs were extracted around the stimulus onset time from −50 to 225 ms using a ±25 μV artifact rejection criterion (Skoe & Kraus, 2010). The EEG recording blocks with more than 10% of the rejected sweeps per block (i.e., >100 rejections) were not included in further analyses (Maggu et al., 2016, 2018). Thus, the minimum number of trials per block was 900. One block comprised the EEG responses to one tone-type for a particular subject at a particular age. The averaged FFR with more than 0.7 μV maximum were excluded for suspicion of stimulus artifact based on preliminary data screening. These procedures resulted in exclusion of 18% of the EEG blocks, including the whole set of data from one participant. In addition, 13% Native /ga2/ blocks, 7% Native /ga4/ blocks, and 9% of the Non-Native /ga4/ blocks were missing in the final data set. The final number of trials per block was 988.8 ± 1.5 for Native /ga2/, 990.0 ± 5.17 for Native /ga4/, and 984.8 ± 2.0 for Non-Native /ga3/. Neither the difference between the tones for the number of accepted trials nor the correlation between the number of trials with age was significant (Native /ga2/ vs. Native /ga4/: *t*(316) = −0.243, *p* = 0.808; Native /ga2/ vs. Non-Native /ga3/: *t*(313) = 1.597, *p* = 0.111; Native /ga4/ vs. Non-Native /ga3/: *t*(323) = 0.981, *p* = 0.327; Native /ga2/ vs. age: *r* = 0.15, *p* = 0.063; Native /ga4/ vs. age: *r* = 0.097, *p* = 0.215; Non-Native /ga3/ vs. age: *r* = 0.054, *p* = 0.5).

FFR was calculated as a time-domain average of all good epochs with baseline correction across the pre-stimulus interval (−50 to 0 ms).

FFR pitch contour was extracted with a sliding time window applied at the time-frequency decomposition of intertrial phase coherence (a.k.a., phase-locking factor, Tallon-Baudry et al., 1996). In comparing FFR pitch contour with auditory stimulus pitch contour, the latter was shifted 20 ms toward the end of the time course for the pitch tracking accuracy calculation (see Jeng et al., 2013 and SI for details).

A number of time-domain and frequency-domain metrics were extracted from FFR. For the main report on FFR here, we selected the four measures most commonly employed in FFR developmental studies: lower-frequency and middle-frequency spectral band-power (Anderson et al., 2012, 2015; Skoe et al., 2015), pitch strength (Jeng et al., 2011, 2013, 2016) and signal-to-noise ratio (SNR). Kolmogorov-Smirnov tests confirmed the normality of the four measures in question (see SI). The FFR SNR was calculated as a dB-transformed ratio between root mean square power of post-stimulus and pre-stimulus intervals of FFR (see SI for the formula). Whole-epoch autocorrelation was calculated on FFR waveforms. Pitch strength was calculated as peak-to-peak distance on an autocorrelation curve between the first autocorrelation peak and the preceding trough. Lower and middle-frequency spectral band-powers were obtained by calculating the fast Fourier transform of FFR waveforms and extracting the power around the F0 and its two lowest overtones (2D and 3D harmonics). See SI and Supplementary Figure 3 for the description of the other metrics.

The aforementioned results focused mostly on the encoding of individual tones. One important requirement of phonological development is to differentiate distinct speech sound categories. We therefore examined classification of the three tones with a support vector machine (SVM) for each month of age that delivered more than 10 observations, i.e., from birth to 14 months. Averaged FFRs were down-sampled to 120 Hz in order to reduce the number of features in the models. The interval from 0 to 150 ms was extracted, resulting in 13 time points/features per observation. The data at each time point were normalized across observations. The data of each age were bootstrapped 10,000 times. In each bootstrap sample, we ran SVM classification with cross-validated accuracy as the outcome. The procedure was performed for both real tone labels and randomly permuted labels labels. (See SI for the detailed SVM parameters.)

### LLR Analysis

For LLR extraction, the data were filtered with a 4-order Butterworth 0.1–30 Hz bandpass filter and down-sampled to 3 kHz. The epochs were extracted around the stimulus latency time from −100 to 600 ms using a ±100 μV artifact rejection criterion. All epochs with amplitude in the artifact rejection criterion were considered good epochs. Since LLR is known to habituate with the repetition of the same sound (Cortesa et al., 2019; Näätänen & Picton, 1987), only 300 first good epochs for each participant/condition were used for the purposes of analysis. LLR was calculated as a time-domain average with baseline correction across the pre-stimulus interval (−100 to 0 ms). SNR was calculated on an averaged LLR with a pre-stimulus interval from −100 to 0 ms and a post-stimulus interval from 0 to 600 ms (see SI for the formula). The LLR main positive peak (i.e., P1) was searched for automatically as a maximum across the post-stimulus interval (0 to 600 ms; see SI for details). Similarly to the FFR analyses, we employed linear mixed-effecs (LME) models with age and tone as fixed effects and participant as a random intercept.

Previous research on the cortical development of lexical tones has demonstrated that 6 months of age is an important milestone for adultlike responses, as the polarity of MMN switches from a positive to a negative (adultlike) response during this period (Cheng et al., 2013). This finding converges with the studies of vowels and consonants that have shown a decrease in MMN responses to non-native sounds in infants older than 6 months of age (Cheour et al., 1998; Rivera-Gaxiola, Silva-Pereyra, & Kuhl, 2005). We tested cortical development of lexical tones by examining age-dependent effects categorically, using 6 months (165 days) as a cutoff. We took a step further to analyze the effects of nativeness by categorizing our stimulus into native (pooled Native /ga2/ and Native /ga4/) and non-native (Non-Native /ga3/) tones with a focus on the latency and amplitude measures. Because P1 peak latency and amplitude did not meet the normality assumption, we used non-parametric tests to investigate the hypothesis of age-dependent sensitivity to speech nativeness.

### FFR vs. LLR Comparison

In order to compare the magnitude of growth between the early-latency neural encoding FFR and long-latency cortical LLR measures, we examined three measures that can be obtained in both FFR and LLR, namely, SNR, amplitude, and latency. We examined a group of younger (*n* = 22) and older (*n* = 17) children with an arbitrary age cutoff of younger than 42 days and older than 400 days, as that would provide the maximum number of participants in each age group. Extracting the effect size of age cutoff (i.e., the slope of the regression line between the two age groups) on the normalized metrics enabled us to compare the maturation of the metrics directly, regardless of individual means and variance, where maturation was indicated by larger SNR, increased amplitude, and shorter latency.

## RESULTS

### Early-Latency Neural Encoding FFR Measures

**Figure 2.**
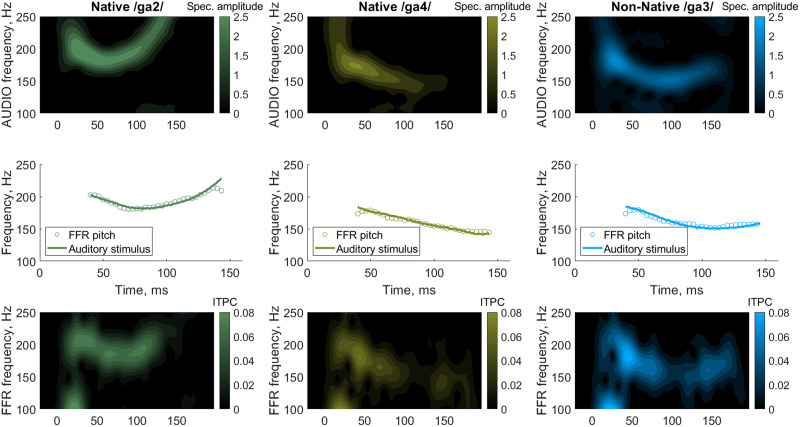
The pitch contour of the frequency-following response (FFR) follows the pitch contour of the auditory stimuli. The columns correspond to the lexical tones Native /ga2/, Native /ga4/, and Non-Native /ga3/. *Upper row*: spectrogram of the auditory stimuli. *Middle row*: the overlap of the auditory (black line) and early-latency brain-response (grey circles) pitch contours. The pitch tracking (audio vs. brain signal correlation) is 0.91 for Native /ga2/, 0.97 for Native /ga4/, and 0.95 for Non-Native /ga3/. *Bottom row*: Grand-averaged time-frequency decomposition of the intertrial phase coherence.

The FFR consists of a series of transient peaks followed by a steady state response (Figure 1, left panel). We first examined whether, on average, the infants’ FFRs showed high fidelity. For each tone, we calculated the intertrial phase coherence extracted pitch contour, which followed the pitch contour of the stimuli when the time lag equal to the averaged latency of stimulus-response correlation (20 ms) was introduced. We found that the averaged pitch contours were strongly correlated with the pitch contours of the speech stimuli, with *r* ^2^ values of 0.91 for Native /ga2/, 0.97 for Native /ga4/, and 0.95 for Non-Native tones (Figure 2). This suggests that as a group, our infant participants were quite accurate in encoding the speech stimuli.

#### LME analysis

**Figure 3.**
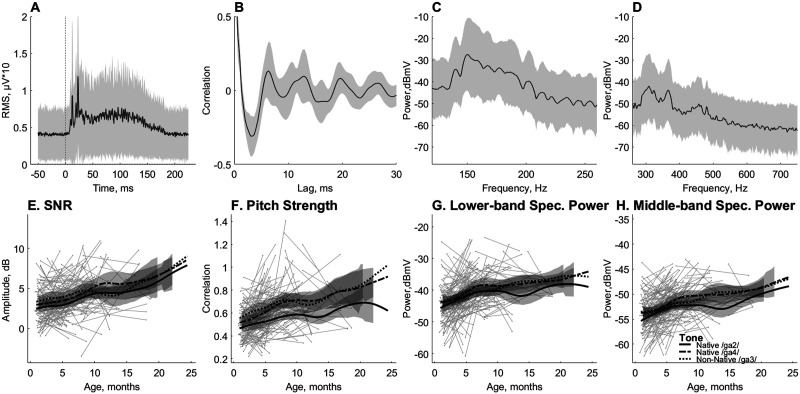
Developmental trajectories of early-latency brain measures show linear growth with age, regardless of speech nativeness. The top panels show the root mean square (RMS) of the frequency-following response (FFR) (A); the FFR autocorrelogram (B); and the lower (120–260 Hz) (C) and middle (260–750 Hz) (D) frequency bands of the FFR spectrum. The shaded area marks the standard deviation. The bottom panels plot the output metrics as a function of age in days. The metrics are the signal-to-noise ratio (SNR) of the time-domain waveform (E); the pitch strength (trough-to-peak magnitude of the autocorrelogram) (F); and the mean power in the lower (G) and middle (H) frequency bands. Individual data points are plotted as grey dots with lines connecting the data of the same participant and the same tone. The three lines represent the average age-dependent development for each of the three lexical tones Native /ga2/ (solid), Native /ga4/ (dash-dotted), and Non-Native /ga3/ (dotted). The data are smoothed with Eilers’ technique (Eilers, 2003).

*F* tests of the fixed effects within the mixed-effects model revealed a main effect of age for FFR SNR (*F*(1, 473) = 19.21; *p* = 1.44 · 10^−5^; *pBf* = 0.0002; η_*p*_^2^ = 0.04), lower-frequency spectral band-power (*F*(1, 473) = 10.91; *p* = 0.0010; *pBf* = 0.0133; η_*p*_^2^ = 0.02), and middle-frequency spectral band-power (*F*(1, 473) = 17.48; *p* = 3.47 · 10^−5^; *pBf* = 0.0005; η_*p*_^2^ = 0.04). The main effect of age on pitch strength was marginally significant (*F*(1, 473) = 7.24; *p* = 0.0074; *pBf* = 0.0958; η_*p*_^2^ = 0.02). There was a marginal effect of tone for pitch strength only (*F*(2, 473) = 4.12; *p* = 0.0169; *pBf* = 0.2198; η_*p*_^2^ = 0.02), while it was not significant for FFR SNR, lower-frequency spectral band-power and middle-frequency spectral band-power (all *p* > 0.05). Most importantly, none of the FFR measures revealed an interaction between age and tone, including FFR SNR, lower-frequency spectral power, middle-frequency spectral power, and pitch strength (all *p* > 0.05, Figure 3).

#### Classification analysis

**Figure 4.**
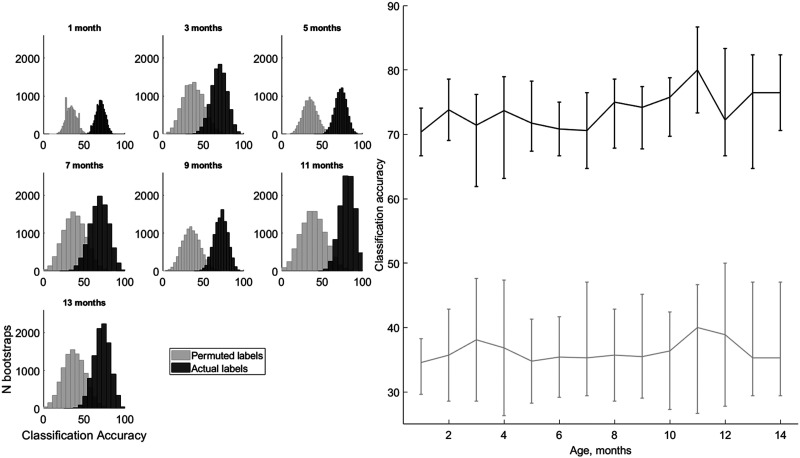
Tone classification with support vector machine improves with age for actual but not for permuted tone labels. The left panel shows real (black) and permuted (grey) labels for the cross-validated classification accuracy distribution across 10,000 bootstrapped samples for selected ages in 2-month steps from 1 to 13 months. The right panel shows the medians of those distributions as a continuous function of age in months from 1 to 14 for real (black) and permuted (grey) labels. The error bars show the range between the first and third quartile of the distributions. The real label classification correlates significantly with age (*r* = 0.64, *p* < 0.05, *d* = 1.67), while the permuted label classification does not (*r* = 0.24, *p* > 0.1, *d* = 0.49).

We found that SVM classification of the early-latency brain responses to the three tones improved over time (Figure 4). The median classification of the tone was overall higher than the permuted classification accuracy (mean 73.8 ± 0.75% and 36.3 ± 0.43%, *t*(13) = 53.48; *p* = 1.25 · 10^−16^; *d* = 14.3). Importantly, classification accuracy improved with age as indicated by the Pearson correlation (*r* = 0.64; *p* = 0.0136; *pBfr* = 0.0272; *d* = 1.67). Adding a quadratic term did not improve the model, suggesting a linear dependency (*F*(1, 11) = 0.14; *p* = 0.71; η_*p*_^2^ = 0.01). No age dependency was found for the permuted data classification (*r* = 0.24; *p* = 0.407; *pBfr* = 0.814; *d* = 0.49).

### Long-Latency Cortical LLR Measures

**Figure 5.**
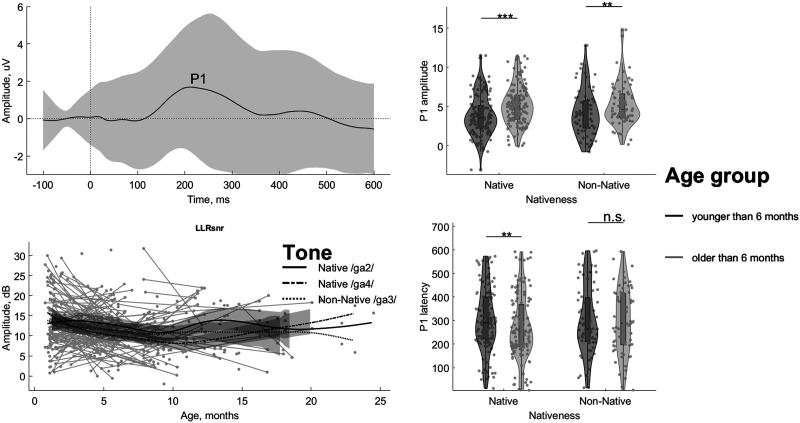
The long-latency response (LLR) signal-to-noise ratio (SNR) does not grow with age, while its peak latency reduces only for the native tones. Upper left panel shows the LLR time-domain waveform averaged across all data points, with the shaded area marking the standard deviation. Peak P1 is labeled at maximum. Lower left panel shows LLR SNR as a function of age in days. Individual data points are plotted as grey dots with lines connecting the data of the same participant and the same tone. The three lines represent the average age-dependent development for each of the three lexical tones Native /ga2/ (solid), Native /ga4/ (dash-dotted), and Non-Native /ga3/ (dotted). The data are smoothed with Eilers’ technique (Eilers, 2003). Upper and lower right panels show violin plots of P1 amplitude and latency distributions across the 6-month cutoff. The tones are pooled into native (/ga2/ and /ga4/, dark-grey) and non-native (/ga3/, light-grey) groups. The grey circles represent individual data and the diamonds mark the medians of the distribution. The significance of the age effect in the Wilcoxon rank-sum test is illustrated with asterisks separately for native and non-native tones (**p < 0.01, ***p < 0.001, uncorrected, n.s. non-significant).

The LLR was dominated by a large and wide positive peak P1 (Figure 1, right panel). We measured the latency and amplitude of P1, as well as its SNR. As in the case of FFR, we investigated the changes of LLR measures as a function of age (Figure 5). In the LME results, there was an increase with age in P1 amplitude (*F*(1, 409) = 9.14; *p* = 0.0027; *pBf* = 0.0345; η_*p*_^2^ = 0.02), but not in LLR SNR (*p* > 0.05) or P1 latency (*p* > 0.05). None of the LLR parameters were different between tones, i.e., LLR SNR, P1 amplitude, or P1 latency (all *p* > 0.05). There was no interaction between age and tones for any of the LLR parameters either, i.e., LLR SNR, P1 amplitude, and P1 latency (all *p* > 0.05). In general, the effect of age on LLR is much less robust compared with the FFR results.

We then followed the findings of the literature concerning the cortical development of lexical tones at 6 months of age (Cheng et al., 2013). For non-native speech, the age split resulted in 63 participants (71 data points) and 51 participants (68 data points), respectively, in the younger and older groups in the analysis. For native speech, 65 participants (135 data points for pooled tones, 75 data points for averaged tones) and 61 participants (141 pooled tone data points, 89 averaged tone data points) were in the younger and older groups, respectively. The Wilcoxon rank-sum tests demonstrated a significant shortening of P1 latency and an increase in its amplitude after the age of 6 months for the native tones (pooled Native /ga2/ and Native /ga4/: *Z* = −2.45, *p* = 0.014, *r* = −0.15 for latency and *Z* = 4.04, *p* = 5.27 · 10^−5^, *r* = 0.24 for amplitude; averaged Native /ga2/ and Native /ga4/: *Z* = −2.41, *p* = 0.016, *r* = −0.19 for latency and *Z* = 3.87, *p* = 1.07 · 10^−4^, *r* = 0.30 for amplitude), while the change was relatively small for the Non-Native /ga3/ tone (*Z* = 0.16, *p* = 0.56, *r* = 0.01 for latency and *Z* = 2.52, *p* = 0.006, *r* = 0.21 for amplitude) (Figure 5, right panel). Overall, these results replicated earlier findings that perceptual narrowing is supported by cortical processes (Conboy & Kuhl, 2011). Specifically, our results here confirmed that sensitivity to the nativeness status of lexical tones occurs at about 6 months of age, as suggested by a previous study (Cheng et al., 2013).

### Early-Latency Neural Encoding FFR vs. Long-Latency Cortical LLR Measures

**Figure 6.**
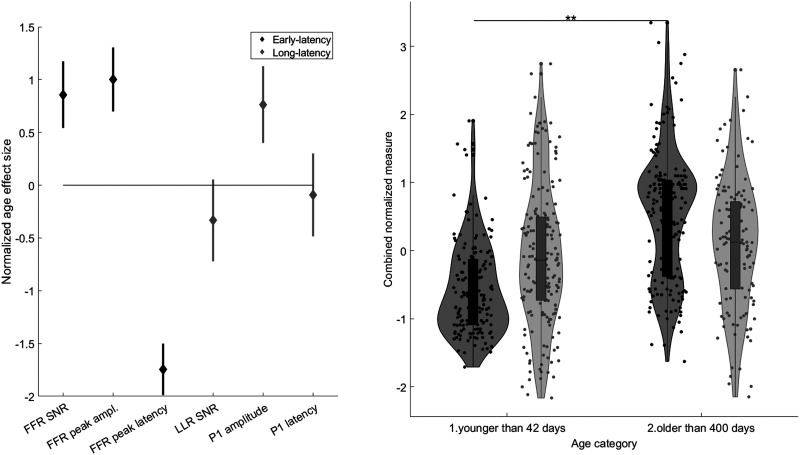
The maturational effect is greater for early-latency (black) than long-latency (grey) response measures. *Left panel*: Both brain subdivisions are represented by three compatible time-domain measures; signal-to-noise ratio (SNR), peak amplitude, and peak latency. The diamonds represent effect sizes measured as the difference between the means of the youngest (younger than 42 days) and oldest (older than 400 days), with participants’ data normalized for each measure. Lines cover the confidence intervals of those effect sizes in the linear models. The confidence intervals for SNR and peak latency do not overlap between the frequency-following response (FFR) and the long-latency response (LLR), indicating significant differences. *Right Panel*: The linear mixed effects analysis shows that a combination of normalized measures grows with age for early-latency (black) but not for long-latency (grey) response measures (**p < 0.01).

As shown in the left panel of Figure 6, the effect size of FFR SNR (0.86, 95% CI [0.54, 1.17]) was significantly larger than that of LLR SNR (−0.33, 95% CI [−0.72, 0.05]). The effect size of FFR peak amplitude (1.00, 95% CI [0.70, 1.30]) was marginally larger than that of LLR P1 amplitude (0.76, 95% CI [0.40, 1.13]). Finally, the effect size of FFR peak latency was significantly larger (i.e., more negative, −1.74, 95% CI [−1.99, −1.50]) than that of LLR P1 latency (−0.09, 95% CI [−0.48, 0.30]). In other words, two out of the three cortical measures showed less change over time, and two out of the three measures showed a significantly larger age-related improvement in the early-latency neural encoding responses.

In order to simultaneously compare the effects of age category on the multiple parameters of early- and long-latency responses, we entered the aforementioned normalized metrics into a combined LME analysis with Age category, Tone, ERP type (early-latency vs. long-latency responses), and ERP measure (SNR vs. latency vs. amplitude) as fixed effects and Subject as random intercept. *F* tests of the fixed effects demonstrated the significant main effect of Age category (*F*(1, 620) = 9.92, *p* = 0.0017; η_*p*_^2^ = 0.02) and the interaction between Age category and ERP type (*F*(1, 620) = 5.62, *p* = 0.0174; η_*p*_^2^ = 0.01). None of the other main effects or interactions was significant (see Supplementary Table 3). In the follow-up split-by-ERP-type LMEs, the metrics of the early-latency (*F*(1, 317) = 10.6, *p* = 0.0013; η_*p*_^2^ = 0.03), but not of the long-latency responses (*F*(1, 303) = 0.018, *p* = 0.8930; η_*p*_^2^ < 0.001), were improved by age (right panel of Figure 6).

## DISCUSSION

The goal of the present study was to investigate the contributions of early-latency neural encoding and long-latency cortical processing toward early language development in the first two years of life. We postulated that a degree of encoding accuracy of speech sounds regardless of native status must occur in the early stages of life, before these speech sounds’ native status could be determined. Consistent with this hypothesis, we found that the early-latency neural encoding of speech grows linearly and markedly for both native and non-native tone categories for children who are learning a tone language. In contrast, the growth in the long-latency cortical responses is much slower, but crucially the change is sensitive to the nativeness status, with changes occurring at about 6 months of age, as reported in previous studies (Cheour et al., 1998; Cheng et al., 2013; Rivera-Gaxiola, Silva-Pereyra, & Kuhl, 2005). We argue that our results reflect the complementary roles of neural structures that are associated with earlier and later stages of processing along the ascending auditory neural pathway. For the frequencies that we examined, these neural structures are likely located in the brain stem and cortex for earlier and later stages of encoding and processing, respectively. While the brain stem (and potentially other neural structures) encodes speech sounds faithfully in an earlier stage of processing, the cortex assigns phonological information to them at a later stage. These two processes together are foundational to early language development.

Behavioral studies of lexical tones suggest that perception of lexical tones emerges at or before 6 months of age for tone-learning infants (Mattock & Burnham, 2006; Yeung et al., 2013). However, it is unclear how development of lexical tone perception is associated with early-latency and long-latency neural responses. As far as we are aware, the present study is the first to address this question. Our long-latency cortical findings are largely consistent with those of the studies of vowel and consonant perception (Cheour et al., 1998; Rivera-Gaxiola, Silva-Pereyra, & Kuhl, 2005) showing native and non-native differentiation in MMN responses after 6 months (and sometimes earlier). Although Cheng et al. (2013) did not investigate native and non-native tones, their findings of an adultlike cortical MMN response to native Mandarin tones in 6-month-old Mandarin-learning infants are generally consistent with the results in the present study. The MMN paradigm favored by the majority of previous LLR studies amplifies the brain responses to the phoneme categories by subtracting standards from deviants in oddball sequence (Escera, 2017; Näätänen & Winkler, 1999). However, in adults the sensitivity to phonological contrasts can be found already in the so-called obligatory brain responses (Grimaldi, 2018). The amplitude of responses to native phonemic contrast was found to increase with age in an equiprobable ERP paradigm (Key et al., 2012). We extended the existing MMN studies by investigating the difference between the obligatory P1 response in infant LLR to stimuli in all-standard blocks. The results converge with the MMN findings as we found a latency delay for non-native tone that is likely to be caused by difficulty in categorization after attunement to native phonology at around 6 months. It is clear that multiple cortical LLR components are responsive to the native status of speech sounds. We must acknowledge that the conclusions derived from our cortical results were based on conducting less robust statistical analyses separating the native and non-native data, rather than relying on findings from a significant Age × Nativeness interaction. Notwithstanding that, the results converged with previous studies.

In terms of early-latency speech encoding processes, Jeng et al. (2013, 2016) were the first to study FFRs in tone-learning infants. These two smaller-scale studies found that neonates from Mandarin-speaking families did not differ from neonates from English-speaking families in their FFRs to Mandarin tones. This suggests the cross-language differences in FFRs to lexical tones found in studies of adults (Krishnan et al., 2010) are most likely attributable to language experience. It should be noted, however, that the developmental time course of FFR to lexical tones and the potential within-subject differences between native and non-native lexical tone encoding have not been investigated before. Two other studies specifically focused on the development of the FFR signal in early years and across the lifespan by examining non-tone language-speaking listeners, but they did not examine lexical tones or the developmental difference between native and non-native speech perception (Anderson et al., 2015; Skoe et al., 2015).

Neurophysiological data suggest that the roles of the brain stem and cortex and the relationship between them may change considerably as the infant grows. Evolutionary older brainstem structures take shape earlier and mature faster than the neocortex (Moore & Linthicum, 2007). Postmortem fetal studies show active processes of axon growth, dendrite tree arborization, and myelination in the brain stem, which start in the last prenatal month and are mostly completed by the end of the first postnatal year (Hartley & King, 2010; Moore et al., 1995, 1997, 1998; Moore & Linthicum, 2007; Sano et al., 2007). It is the neocortex that empowers humans with speech, but the development of cortical structure achieves its adult-like condition only by the age of 11–12 years (Moore & Guan, 2001). Indeed, the functioning of the auditory cortex in the perinatal period is physiologically very different from its functioning in adults in terms of the cortical layer domination (Moore & Linthicum, 2007). The immature status of the cortex implies also the immature condition of the corticofugal projections to the brain stem. Thus, the potential contribution of the corticofugal system to speech perception in adults may be related to its maturation (Suga et al., 2000). On the other hand, recent studies have clearly shown that the adult midbrain is not a mere relay and is capable of detecting regularities in the auditory stream (Malmierca et al., 2019). This capability also needs time to mature. Our data confirm previous descriptions using non-invasive EEG recordings. We have followed several non-invasive measures of early-latency neural encoding that are likely to reflect the spread of myelination and increase in synaptic activity in the brain stem and structures connected to it. Moreover, we show that cortical responses grow more slowly and that their maturation is also native-language specific. We anticipate that further studies will combine functional methods such as EEG with non-invasive structural methods such as diffusion MRI in order to obtain a better understanding of the neurophysiological basis of speech acquisition.

Neural phase-locking to the stimulus envelope is the most accurate representation of the physical properties of the stimulus. It requires accurate timing of neural firing, i.e., a strong degree of myelination. The capability of neurons in phase-locking to the high frequencies reduces along the auditory axis, and in the cortex the spatial coding of frequency (tonotopy) is prevalent instead (Leaver & Rauschecker, 2016). The FFR may correspond to the phase-locked activity of neurons of different neural centers across the ascending auditory pathway. The empirical studies available to date seem to suggest that phase locking at higher frequencies (>150 Hz) in the FFR most likely originates from the auditory brain stem (Bidelman, 2018; Tichko & Skoe, 2017). The basic conceptualization proposed here is not restricted to the brain stem or another particular neural structure per se, but rather suggests a division of labour of different auditory neural centers during language development. It seems likely that some centers contribute to representing basic acoustic elements of speech via direct phase-locking to the stimulus, while others assign function to those acoustic elements. They both need to be in place in order for spoken language to develop. It is worth emphasizing that our present findings do not inform—nor do they depend on—our understanding of the precise neuroanatomical origins of the early-latency neural speech encoding. Our results merely suggest that not all neural responses to speech are equal in their neurodevelopmental trajectories, and most importantly the differences between temporally earlier FFR and later LLR are important for a child to succeed in acquiring native speech. Future research should examine the exact locations of the neural generators of these two types of responses.

We studied lexical tone and speakers of one tone language as the starting point to investigate the development of FFR and LLR in relation to native and non-native speech sounds. The broader applicability of these findings must be ascertained through future studies of other speech categories and young children learning other languages. Future research must also consider the impact of sleep on neural measurements. Although the vast majority of our participants were sleeping naturally during the experiment, it was not clear at which exact stage of the sleep cycle, and a few were even awake during the EEG recording. Research has demonstrated that different sleep stages may affect the amplitude of the LLR, specifically P3 at the latency of around 600–700 ms (Barnet et al., 1975). Although the focus of our LLR analysis was on the P1 component, we could not completely rule out the influence of sleep stage on our results. We expected that our large sample size and recordings at multiple time points would reduce the impact of sleep on our findings. Nonetheless, we must acknowledge that the lack of information about sleep stages from our participants might have affected the results. Therefore, an important future direction of this line of research is to establish to what extent our results are reproducible in the fully awake infants.

In summary, our findings suggest that neural centers at the earlier stages of the ascending auditory pathway are crucial for native language development because they provide an accurate representation of an incoming acoustic signal, regardless of whether or not those sounds are native. This accurate representation would allow neural centers at later stages of the auditory pathway to determine their linguistic relevance. Our study provides a critical piece of information about language development that, to the best of our knowledge, no previous study has considered. It opens up a new line of inquiry for future research. For example, future studies should examine the minimum required encoding accuracy in the brain stem and other structures before the signal is sufficiently accurate for linguistic processing in the cortex. They should investigate the age at which early-latency neural encoding contributes to language development when the child is older, and the extent of this contribution. Given that long-term sound exposure can modulate early-latency neural encoding in adulthood (Krishnan et al., 2005, 2010; Wong et al., 2007), future studies should examine the age at which the influence of auditory experience on neural encoding of native speech emerges. In terms of clinical application, an understanding of the division of labour among different centers of the auditory pathway would enable diagnostic protocols to be developed for investigating different subtypes of central auditory processing and language problems (Ingvalson & Wong, 2013; Ettlinger et al., 2014). While some disorders may be a result of poor encoding at lower-level centers, others may stem from a failure to assign accurate phonological categories in the cortex.

## ACKNOWLEDGMENTS

The authors wish to thank the families for participating in this study, many of whom were participants in the Stanley Ho Developmental Cohort Study. We would also like to thank Lydia Leung, Agnes Chou, Debby Ma, and Charlene Chiu for their assistance. This work was supported by the Dr. Stanley Ho Medical Development Foundation and a grant from the Innovation and Technology Fund (Hong Kong) #ITS/067/18. Some of the results reported in this article were presented at the 2019 Annual Meeting of the Organization for Human Brain Mapping.

## FUNDING INFORMATION

Patrick Wong, Innovation and Technology Commission - Hong Kong (https://dx.doi.org/10.13039/501100007156), Award ID: ITS/067/18. Patrick Wong, Dr. Stanley Ho Medical Development Foundation.

## AUTHOR CONTRIBUTIONS

**Nikolay Novitskiy**: Data curation, Formal analysis, Software, Validation, Visualization, Writing – original draft. **Akshay R. Maggu**: Conceptualization, Methodology, Investigation, Writing – review & editing**. Ching Man Lai**: Data curation, Investigation, Project administration, Writing – review & editing. **Peggy H. Y. Chan**: Data curation, Investigation, Writing – review & editing. **Kay H. Y. Wong**: Data curation, Investigation, Writing – review & editing. **Hugh Simon Lam**: Supervision, Resources, Writing – review & editing. **Tak Yeung Leung**: Resources, Writing – review & editing**. Ting Fan Leung**: Supervision, Resources, Writing – review & editing. **Patrick C. M. Wong**: Supervision, Conceptualization, Methodology, Project administration, Funding acquisition, Resources, Writing – original draft.

## COMPETING INTERESTS

Patrick C. M. Wong is the founder of a startup company supported by a Hong Kong SAR Government startup scheme for universities.

## TECHNICAL TERM

Frequency-following response (FFR): Scalp recordings of human phase-locked neural activity that are synchronized to periodic and transient aspects of sound.
