# Supplementary material for: Early Development of Neural Speech Encoding Depends on Age but Not Native Language Status: Evidence From Lexical Tone
Source: Neurobiol Lang (Camb). 2022 Feb 10;3(1):67–86. doi: 10.1162/nol_a_00049 (PMC10178623; doi:10.1162/nol_a_00049)
Supplement: Supplementary file 1 [file nol-3-1-67-s001.docx]

**SUPPLEMENTARY INFORMATION**

**Materials and Methods**

1. **Participants**

All participants were healthy infants and toddlers who had no reported genetic abnormalities, required no resuscitation or help with breathing at birth and had not been diagnosed with any other medical problems either at birth or after birth. The minimal gestational age at birth was 37 weeks and minimal weight at birth was 1.8 kg. Families were recruited via the news media from different parts of Hong Kong as well as from our primary teaching hospital. Initial testing was conducted when the participants were aged between 1 and 17 months. After the initial testing, they were asked to come back within the next 5 to 25 months for additional testing, at a time convenient to them. They were free to come back as many times as they wished within this interval (**Supplementary Fig. 2**). The final set included 28 one-month-olds, 15 two-month-olds, 8 three-month-olds, 7 four-month-olds, 18 five-month-olds, 19 six-month-olds, 7 seven-month-olds, 11 eight-month-olds, 11 nine-month-olds, 14 ten-month -olds, 7 eleven-month-olds, 7 twelve-month-olds, 7 thirteen-month-olds, 6 fourteen-month -olds, 2 fifteen-month-olds, 5 seventeen-month-olds, 3 eighteen-month-olds and a child per each of the 19, 20, 21, 23, 24 and 25-month-old categories. Note that the total size of the set is higher than the number of individual subjects because each age of a longitudinal subject produced an additional entry in the set.

The experiments were scheduled around the natural nap time of the participants. For the vast majority (95%), the child was naturally sleeping in the arms of the caregiver during the EEG recording. Because insert earphones were used, the caregiver could not hear the speech stimuli. The experimenter was able to see the caregiver and child through a window of the recording chamber. About 5% of the participants could not fall asleep and the EEG recordings were made while they were awake.


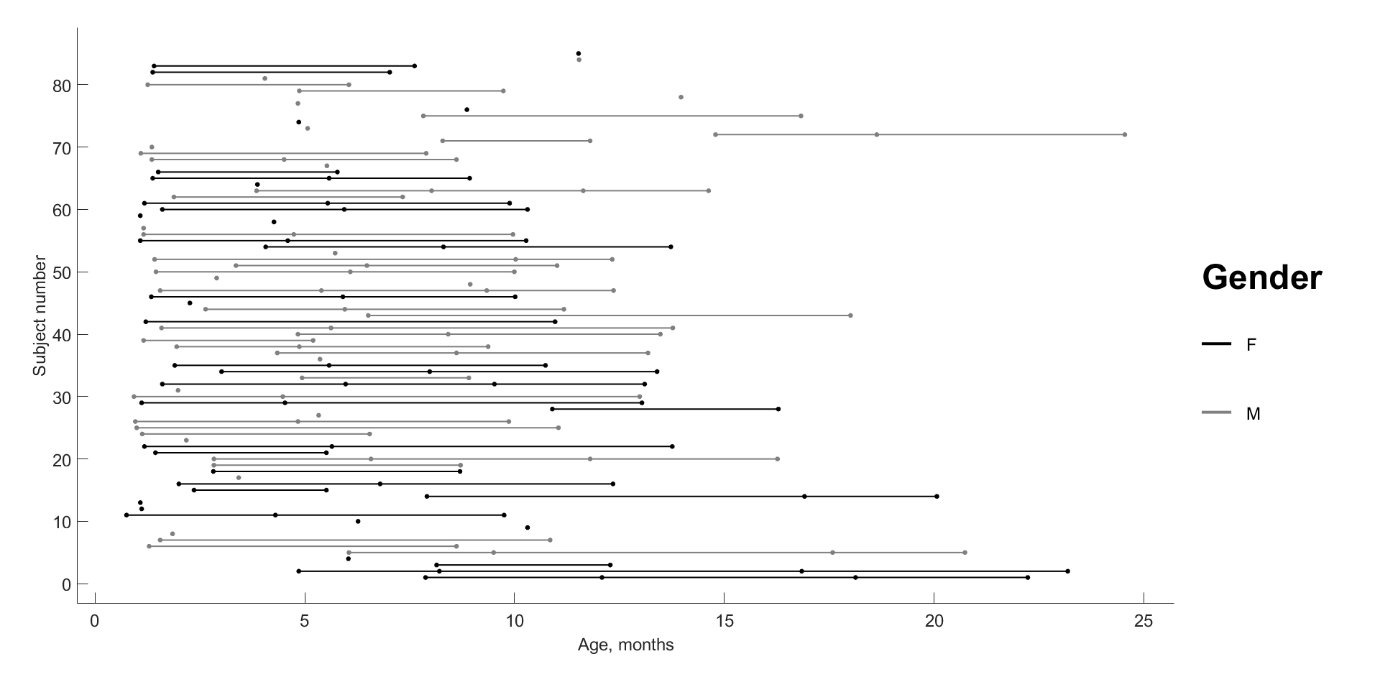


**Supplementary Figure 2.** Most of the participants were tested longitudinally (56 out of 85). The x-axis indicates the age of recording and the y-axis plots unique participant numbers. The data of the same participant are connected by lines. Male data points are grey (N=48), female data points are black (N=37).

1. **Stimuli**

The **Supplementary Figure 1** illustrates the temporal and spectral characteristics of the stimuli. The Euclidean distances between pairs of tone contours were: 54 Hz (Native /ga2/ - Native /ga4/), 48 Hz (Native /ga2/ - Non-Native /ga3/) and 8 Hz (Native /ga4/ - Non-Native /ga3/). The F0 difference between the two Cantonese tones Native /ga2/ and Native /ga4/ was therefore larger than that between either of them and the Mandarin Non-Native /ga3/ tone. The complexity of the tone was estimated as the curvature (C) of their pitch contour (Pc) along the time-course (Tc) with the formula

$$C=\frac{Tc^{'}\times Pc^{''}-Pc^{'}\times Tc^{''}}{{\sqrt{{Tc}^{'2}+{Pc'}^{2}}}^{3}}$$

where Tc’, Pc’ and Pc’’, Tc’’ are the first and second derivatives with respect to sampling points. This method of quantification is more objective than visual inspection. The mean C across the time-course was 0.0008 for Native /ga2/, 0.0035 for Non-Native /ga3/ and 0.0015 for Native /ga4/. Thus the contour of Non-Native /ga3/ was the most complex of the three tones geometrically. However, as discussed in the Results section below, the accuracy of encoding of Non-Native /ga3/ was better than that of Native /ga2/. Thus, acoustic complexity is unlikely to be a relevant consideration in our overall interpretation of the results.

**Supplementary Figure 1.** The Chinese lexical tones embedded in the same syllable can be clearly distinguished in frequency domain. *Left column***:** waveforms of the three lexical tones Native /ga2/ (upper row), Native /ga4/ (middle row) and Non-Native /ga3/ (lower row). *Middle column:* the spectrograms of the three lexical tones in the frequency range from 50 to 1000 Hz in the same order: Native /ga2/ (hunter green), Native /ga4/ (olive green) and Non-Native /ga2/ (blue). The extracted pitch contour is plotted as a white dashed line. *Right column*: comparison between the pitch contours of the tones Native /ga2/ (solid), Native /ga4/ (dashed-dotted) and Non-Native /ga3/ (dotted).

The tones were presented at an intensity of 70 dB SPL while the participant was asleep. Alternating polarity was used to minimize stimulus artifacts and cochlear microphonics (Gorga, Worthington, Reiland, Beauchaine, & Goldgar, 1985; Skoe & Kraus, 2010). Each tone was presented in one block. The block order of the tones was pseudo-randomized between participants, and they were separated with a silent pause. The stimuli were presented binaurally via insert earphones (Etymotic Research, USA). Excluding preparation time, the experiment lasted between 30-35 minutes with all three blocks.

1. **FFR analysis**
   1. Time-domain FFR analysis

**SNR** was calculated as a ratio between RMS power of post-stimulus and pre-stimulus intervals of an averaged FFR response, converted to dB (Anderson, Parbery-Clark, White-Schwoch, & Kraus, 2015; Parks, Gannon, Long, & Young, 2016) :

$$SNR=20\cdot lg\left( \frac{RMSpoststimulus}{RMSprestimulus} \right)$$

**Response consistency** was calculated as a correlation between the FFRs of the subsamples of the original epoch sets. The epochs were split into two randomly selected halves 300 times and each time the FFRs were calculated as averages of each half (Anderson, Parbery-Clark, White-Schwoch, & Kraus, 2012; Skoe, Krizman, Anderson, & Kraus, 2015). The cross-correlation between the two FFRs was calculated and averaged across permutations. Finally, the correlation coefficient r for each participant was Fisher transformed according to the formula:

$$Fisher\left( r \right)=\frac{1}{2}\cdot ln \left( \frac{1+r}{1-r} \right)$$

We detected FFR peak automatically as an absolute maximum in the 2 to 25 ms post-stimulus interval. This positive peak is most likely homologous to the peak V in the peak-picking procedure. The amplitude and latency of this peak were taken into analysis as **FFR peak amplitude** and **FFR peak latency**.

Whole-epoch autocorrelation was calculated with MATLAB function *xcorr*. The sequence was normalized so that the autocorrelations at zero lag are identically 1. The autocorrelogram curve represents a damping oscillation (**Fig. 3B**). For each individual participant and condition, we first found the biggest trough on the curve that occurred after the peak at 0 ms lag and measured its amplitude (autoanticorrelation). The amplitude of the first peak after the first trough was measured as autocorrelation. Adapted from Jeng, Peris, Hu, & Lin, (2013), the difference between peak (autocorrelation) and trough (autoanticorrelation) was calculated and reported as the **pitch strength.**

For stimulus-response correlation calculation, auditory stimuli were imported into MATLAB and downsampled to the response sampling rate of 3000 Hz. The resulting waveform was correlated with the response in a sliding way, sample by sample with MATLAB *xcorr* function. The maximal and minimal value (peak and trough) of the correlation was searched for across the whole length of the curve and recorded as stimulus-response correlation and anticorrelation correspondingly. The difference between stimulus-response correlation and anticorrelation was calculated and reported as **stimulus fidelity**. The latency of the stimulus-response correlation was measured as the stimulus-response correlation delay. The knowledge of the averaged stimulus-response correlation delay proved to be instrumental in pitch tracking accuracy calculation.

- 1. Frequency-domain FFR analysis

The whole-trial spectrum was obtained with ERPLAB toolbox (Lopez-Calderon & Luck, 2014). An FFT in Hanning window was applied to the time-domain average of all good epochs (trials). The resulting FFT spectrum consisted of 8192 points in the 0-1500 frequency range, providing a resolution of 0.1831 Hz. It was converted to dB(mV) by taking the 20·Log_10_ of the value. The averaged **power** was extracted in the **lower** (120-260), **middle** (260-750) and **higher** (750-1200) frequency ranges that approximately correspond to the voice pitch (F0), its two lowest overtones (2d and 3d harmonics), and the higher harmonics that carry the first and second formant of the ‘a’ vowel, respectively. Note that this procedure removes the information about the shape of the pitch contour that is important in tone language.

In order to extract pitch contours, we performed time-frequency decomposition with **ITPC**. ITPC was calculated with EEGLAB 14.1.2 function *newtimef* (Delorme & Makeig, 2004). We used a Morlet wavelet with 166-ms time window, 5 cycles at the lowest frequency, and the 0.01 cycle number increase rate with the frequency. Note that artifactual epochs were not excluded at this stage. The time-frequency decomposition was limited to the female voice pitch range 100 to 250 and resulted in a matrix of 50 frequency points by 200 time points with frequency resolution 3 Hz and mean temporal resolution 1.1 ms. The time range was -22 to 197 ms across the stimulus onset. At each point of the time-frequency matrix, ITPC was calculated as an absolute value of a mean across the trials (epochs) of the ratio between each time-frequency point complex value divided by its absolute value (Delorme & Makeig, 2004). ITPC varies between zero and one, where one means the same phase (angle on the complex plane) of the time-frequency points in all trials (epochs) and zero means a completely random distribution of phases across the trials. Larger ITPC values can be interpreted as stronger phase-locking between the trials, hence the alternative name for ITPC is phase-locking factor (Tallon-Baudry, Bertrand, Delpuech, & Pernier, 1996). ITPC was quantified as absolute maximum across the matrix.

Pitch was extracted from the ITPC matrices according to the following algorithm. First, we calculated the maximum ITPC across the frequencies at each time point. Second, all the points that were smaller than the 50% of the absolute maximum ITPC were excluded. That mostly led to the removal of the pre-stimulus and post- stimulus intervals from the pitch contours. Stimulus pitch was extracted with the same algorithm from the spectrogram of the auditory stimulus. The spectrogram for each of the three auditory stimuli was built with the *spectrogram* function of the MATLAB Signal Processing Toolbox (**Fig. 2** and **Supplementary Fig. 1**). In our data, the FFR followed auditory stimulus with a characteristic delay (presumably due to the neural conduction time) that was on average 20 ms (See Stimulus-Response correlation). The auditory stimulus pitch contours were therefore shifted 20 ms forward in time. **Pitch tracking** **accuracy** was calculated as a squared correlation between the stimulus and response pitch contours, both locally (i.e. for every individual participant) and globally (i.e. for the comparison of the auditory pitch with the grand average pitch contour), and separately for each of the three tones (Jeng et al., 2013).

3.3 Normality test

Normality is a prerequisite of performing many parametric tests. In the case of a multi-factor comparison, we are interested in the normality of the residuals of a particular model. We aimed to analyse the effect of tone and age on each of the EEG parameters. Therefore, we tested the residuals of mixed-effects 2-factor linear models (Tone and Age) for normality with a Kolmogorov-Smirnov (KS) normality test. The KS test produces a p-value that is a probability of obtaining the data sample if the H0 is true. In the KS test the H0 postulates that the sample is taken from a normal distribution. Thus the bigger the p-value of the KS test, the greater the probability that the data is taken from a normal distribution. Thus one of the prerequisites of a parametric test is fulfilled. A conventional p=0.05 cut-off can be used to make a categorical judgement on the normality of the data. The procedure included fitting a linear model for every parameter, extracting the residuals and performing a KS test on the residuals. If the KS test of the residuals confirms the normality, then we turn to the results of the linear model analyses in the form of Linear mixed-effects model, Pearson correlation coefficients. If the KS test fails to confirm the normality, we ignore the linear model results and switch to non-parametric tests, namely Spearman’s rank correlation and the Wilcoxon rank-sum test.

- 1. Statistical tests.

The linear mixed models were calculated with the *fitlme* function of MATLAB’s Statistics and Machine Learning Toolbox. We used the default Maximum likelihood estimation method (ML) and the default coding for dummy variables (‘reference’). The F-statistics for fixed effects within mixed-effects models were quantified in the same MATLAB Toolbox with the *anova* method for linear mixed-effects models, using the default method for computing approximate degrees of freedom (‘residual’).

The effect size of LME F-statistics was calculated as η^2^_p_ (partial eta squared) according to the formula (Lakens, 2013):

$$\eta_{p}^{2}=\frac{F\cdot DF1}{F\cdot DF1+DF2},$$

where F is F-statistics, DF1 is the effect degrees of freedom number and DF2 is the error degrees of freedom number. The effect size of a paired t-test was calculated according to the formula:

$$d=\frac{t}{\sqrt{N}},$$

where t is t-statistics and N is the number of observations where r is the Pearson correlation coefficient. The effect sizes for Wilcoxon tests were calculated as r values according to the formula:

$$r=\frac{Z}{\sqrt{N}},$$

where Z is the Wilcoxon Z-statistics and N is the number of observations. The effect sizes for Pearson correlations and Spearman correlations are their r and ρ values correspondingly. We also provide Cohen’s d for Pearson correlation that was re-calculated from the r-value according to the formula:

$$d=\frac{2r}{\sqrt{1-r^{2}}}$$

- 1. Support Vector Machine (SVM) implementation

The following SVM parameters of the model were chosen in the LIBSVM package (Chang & Lin, 2001): C-SVC type of SVM, radial basis function kernel, gamma of the kernel 1/number of features, cost 100, no shrinking. Note that although the original SVM algorithm had been developed for binary classification, the LIBSVM package (Chang & Lin, 2001) implements multi-class classification with “one-against-one” method (Hsu & Lin, 2002). The median classification accuracy of the tone across ages was compared with the permuted classification accuracy with a t-test. A Pearson correlation of classification accuracy with age was performed for both the actual and the permuted data classification.

1. **LLR analysis**

LLR peak can be detected as absolute maximum across the whole epoch. However, this algorithm might erroneously place the peak at the edge of the epoch when there is either no true maximum within an epoch (amplitude grows or falls monotonously across the epoch) or the actual local maximum has a smaller amplitude than the edge values. The former situation should be excluded in the LLR measurement as a monotonous change would most likely indicate the absence of any physiological response. In the latter situation a true extremum should be found. Therefore, we applied a two-step procedure to detect LLR maxima. First, we located all the true maxima in a post-stimulus epoch (0-600 ms) as the points where the time-course derivative crosses zero into negative values. Then we detected the global maximum as the largest of the local maxima across the post-stimulus epoch. If no extremum was detected at the interval, the time-course was marked as one lacking a P1 peak.

**Supplementary Results**

*Normality assumption check*

As a prerequisite for the maturational effect investigation, we tested for normality the residuals of the 2-factor linear mixed-effects model (Age and Tone as fixed effects and Subject as a random intercept). The following FFR measures showed normality; middle-frequency spectral power (KS p=0.92395), lower-frequency spectral power (KS p=0.52991), higher-frequency spectral power (KS p=0.10308), SNR (KS p=0.66817), FFR peak amplitude (KS p=0.065452), pitch strength (KS p=0.30819), stimulus fidelity (KS p=0.9943), FFR peak latency (KS p=0.77083) and pitch tracking accuracy (KS p=0.067445). The normality of ITPC (KS p=0.0075) was not confirmed. KS tests also confirmed the normality of LLR SNR residuals for mixed-effects models (KS p=0.089055). The residuals of other parameters, namely P1 peak amplitude and P1 peak latency were not normally distributed (KS p<0.05). Parametric tests were only conducted with measures that met the normality assumption, while non-parametric tests were used for those that did not.

*Supplementary LME results*.

Linear Mixed Effects (LME) analysis demonstrated the growth with age for FFR peak amplitude (F(1,473)= 43.51; p=1.13·10^-10^; pBf=1.47·10^-9^; η^2^_p_=0.08), and FFR peak latency (F(1,280)=192.11; p=1.28·10^-33^; pBf=1.67·10-32; η^2^_p_=0.4). The effect was marginal for stimulus fidelity (F(1,473)=4.10; p=0.0435; pBf=0.5659; η^2^_p_=0.009) and was lacking completely for higher-frequency spectral band power and pitch tracking (p>0.05 for all parameters). There was no effect of tone for any of the supplementary FFR parameters, i.e. higher-frequency spectral band power, FFR peak amplitude, stimulus fidelity, pitch tracking, and FFR peak latency (p>0.05 for all parameters). There was no interaction between age and tone for any of the supplementary FFR parameters either, i.e. higher-frequency spectral band-power, FFR peak amplitude, stimulus fidelity, ITPC, pitch tracking and FFR peak latency (p>0.05 for all parameters).

*Linear regression.*

To provide convergence with our LME findings reported above, we also conducted a series of traditional linear regression analyses (**Supplementary Tables 1-2, Supplementary Figures 3-4**).

**Supplementary Table 1.** Results of parametric correlations for measures that met normality criterion. (pBfr – Bonferroni-corrected p-value).

| ERP measure |  | Pearson’s correlation with age | | | |
| --- | --- | --- | --- | --- | --- |
|  |  | Overall | Native /ga2/ | Native /ga4/ | No-Native /ga3/ |
| FFR SNR | R | 0.34 | 0.3 | 0.37 | 0.35 |
|  | P | 7.07·10^-15^ | 2.99·10^-8^ | 6.48·10^-8^ | 0.0023 |
|  | pBfr | 1.00·10^-13^*** | 4.19·10^-7^*** | 9.07·10^-7^*** | 0.0323* |
|  | d | 0.72 | 0.63 | 0.8 | 0.75 |
| FFR pitch strength | R | 0.32 | 0.31 | 0.4 | 0.3 |
|  | P | 6.01·10^-13^ | 9.87·10^-5^ | 1.29·10^-7^ | 1.24·10^-4^ |
|  | pBfr | 8.41·10^-12^*** | 0.0014** | 1.81·10^-6^*** | 0.0017** |
|  | d | 0.68 | 0.65 | 0.87 | 0.63 |
| FFR lower-frequency spectral power | R | 0.31 | 0.26 | 0.32 | 0.34 |
|  | P | 7.82·10^-12^ | 0.0010 | 2.37·10^-5^ | 1.15·10^-5^ |
|  | pBfr | 1.09·10^-10^*** | 0.0143* | 3.33·10^-4^*** | 1.62·10^-4^*** |
|  | d | 0.65 | 0.54 | 0.68 | 0.72 |
| FFR middle-frequency spectral power | R | 0.34 | 0.3 | 0.37 | 0.35 |
|  | P | 3.04·10^-14^ | 1.37·10^-4^ | 5.19·10^-6^ | 7.73·10^-7^ |
|  | pBfr | 4.26·10^-13^*** | 0.0019** | 1.08·10^-5^*** | 7.26·10^-6^*** |
|  | d | 0.72 | 0.63 | 0.8 | 0.75 |
| FFR peak amplitude | R | 0.46 | 0.48 | 0.46 | 0.42 |
|  | P | 5.23·10^-26^ | 4.44·10^-10^ | 1.05·10^-10^ | 2.91·10^-8^ |
|  | pBfr | 7.32·10^-25^*** | 6.21·10^-9^*** | 1.46·10^-9^*** | 4.08·10^-7^*** |
|  | d | 1.04 | 1.09 | 1.04 | 0.93 |
| FFR higher-frequency spectral power | R | 0.11 | 0.12 | 0.12 | 0.1 |
|  | P | 0.0139 | 0.1405 | 0.1278 | 0.1966 |
|  | pBfr | 0.19 | >1 | >1 | >1 |
|  | d | 0.22 | 0.24 | 0.24 | 0.2 |
| FFR stimulus fidelity | R | 0.11 | 0.16 | 0.09 | 0.08 |
|  | P | 0.0184 | 0.0459 | 0.3328 | 0.2734 |
|  | pBfr | 0.26 | 0.6430 | >1 | >1 |
|  | d | 0.22 | 0.32 | 0.18 | 0.16 |
| LLR SNR | R | -0.138 | -0.0607 | -0.2176 | -0.1486 |
|  | P | 0.0047 | 0.4842 | 0.0095 | 0.0809 |
|  | pBfr | 0.0656 | >1 | >1 | >1 |
|  | d | -0.279 | -0.122 | -0.446 | -0.301 |


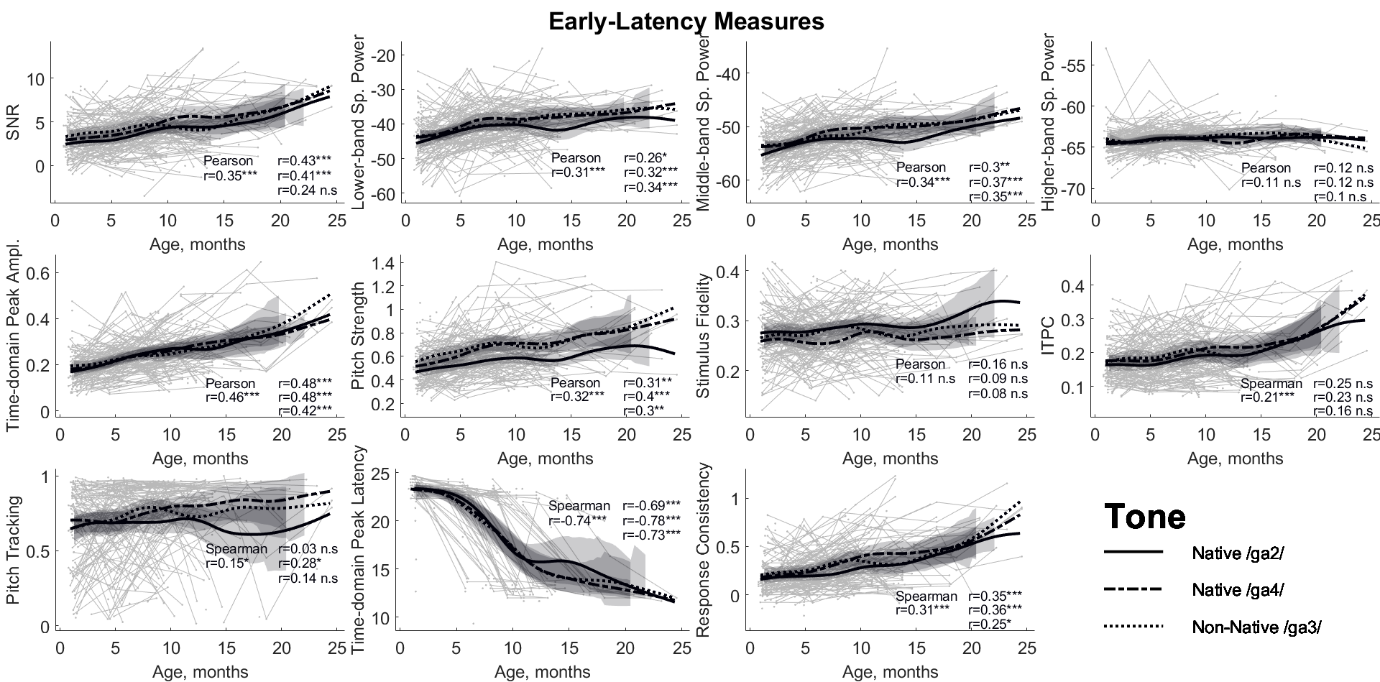


**Supplementary Figure 3.** Developmental trajectories of 11 early-latency neural encoding measures. Each panel shows the plot of the output metrics as a function of age in days. The metrics are lower, middle, and higher-frequency spectral power, stimulus fidelity, signal-to-noise ratio (SNR), FFR peak amplitude and latency, pitch strength, response consistency, inter-trial phase coherence (ITPC), and pitch tracking accuracy. Individual data points are plotted as grey dots with lines connecting the data of the same participant and the same tone. The three lines represent the average age-dependent development for each of the three lexical tones Native /ga2/ (solid), Native /ga4/ (dash-dotted) and Non-Native /ga3/ (dotted). The data are smoothed with Eilers’ technique (Eilers, 2003). Pearson correlation was applied for the measures with confirmed normality and Spearman correlation was used for the other measures. The correlation type and coefficients are indicated on the plot. The correlation coefficients are given for the pooled age effect (left column under the correlation type) and separately by tone (right column, in the following order: Native /ga2/, Native /ga4/ and Non-Native /ga3/). The significance is illustrated by asterisks: *p<0.05, **p<0.01, ***p<0.001, n.s. non-significant, Bonferroni-corrected across 14 measures and, when applicable, 3 tones.

**Supplementary Table 2**: Results of non-parametric correlations for measures that did not meet normality criterion (pBfr – Bonferroni-corrected p-value).

| ERP measure |  | Spearman’s correlation with age | | | |
| --- | --- | --- | --- | --- | --- |
|  |  | Overall | Native /ga2/ | Native /ga4/ | No-Native /ga3/ |
| FFR response consistency | rho | 0.31 | 0.35 | 0.36 | 0.25 |
|  | p | 2.36·10^-12^ *** | 6.30·10^-6^ | 2.44·10^-6^ | 0.0011 |
|  | pBfr | 3.31·10^-11^*** | 0.0003*** | 0.0001*** | 0.0471* |
| FFR peak latency | rho | -0.74 | -0.69 | -0.78 | -0.73 |
|  | p | 2.61·10^-50^ | 1.72·10^-14^ | 4.79·10^-21^ | 1.02·10^-17^ |
|  | pBfr | 3.65·10^-49^*** | 7.17·10^-13^ | 2.01·10^-19^ | 4.27·10^-16^ |
| FFR ITPC | rho | 0.21 | 0.25 | 0.23 | 0.16 |
|  | p | 5.33·10^-6^ | 0.0018 | 0.0031 | 0.0490 |
|  | pBfr | 7.46·10^-5^ *** | 0.0746 | 0.1318 | pBfr>1 |
| FFR pitch tracking accuracy | rho | 0.15 | 0.03 | 0.28 | 0.14 |
|  | p | 0.0012 | 0.6818, | 0.0006 | 0.0790 |
|  | pBfr | 0.0175* | pBfr>1 | 0.0232* | >1 |
| LLR P1 amplitude | rho | 0.276, | 0.25 | 0.31 | 0.27 |
|  | p | 1.13·10^-8^ | 0.0034 | 0.0002 | 0.0013 |
|  | pBfr | 1.57·10^-7^*** | 0.1435 | 0.0094** | 0.0541 |
| LLR P1 latency | rho | -0.081 | 0.08 | -0.13 | -0.04 |
|  | p | 0.099 | 0.3521 | 0.1341 | 0.6086 |
|  | pBfr | >1 | >1 | >1 | >1 |


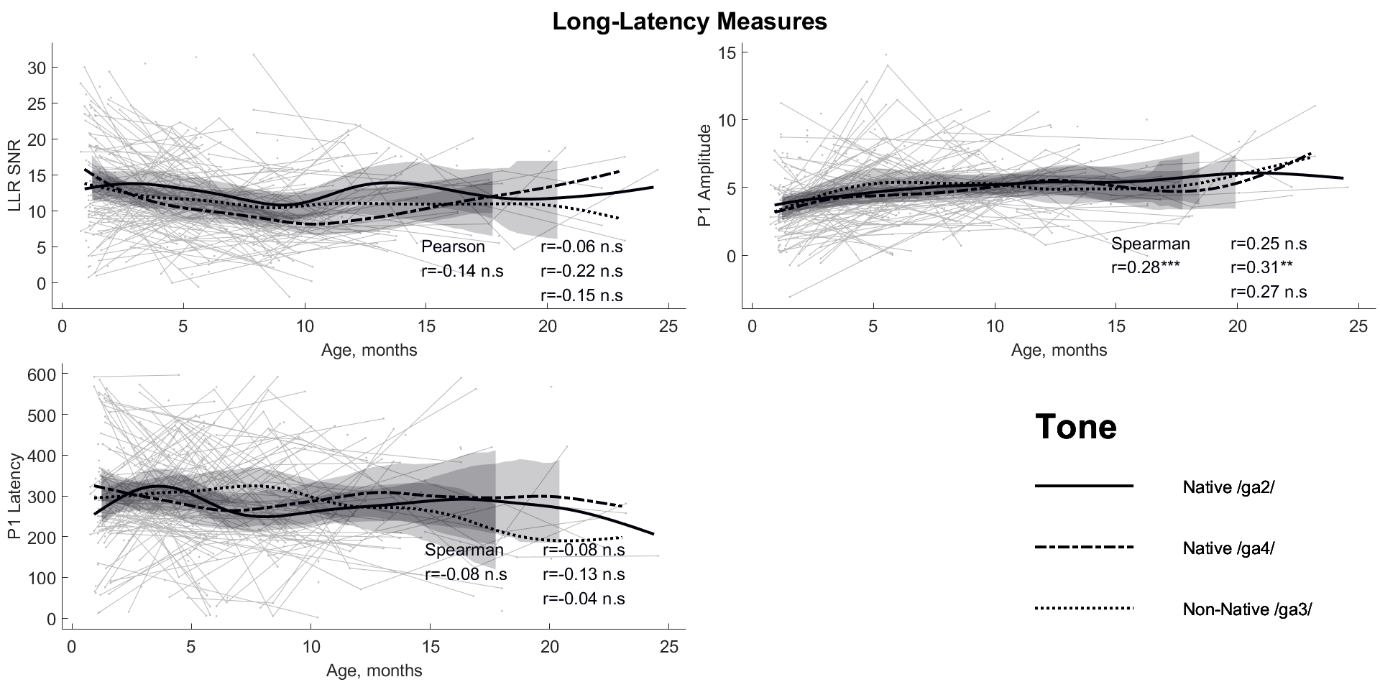


**Supplementary Figure 4.** Developmental trajectories of 3 long-latency cortical measures. Each panel shows the plot of the output metrics as a function of age in days. The metrics are P1 amplitude and latency. Individual data points are plotted as grey dots with lines connecting the data of the same participant and the same tone. The three lines represent the average age-dependent development for each of the three lexical tones Native /ga2/ (solid), Native /ga4/ (dash-dotted) and Non-Native /ga3/ (dotted).The data are smoothed with Eilers’ technique (Eilers, 2003). Pearson correlation was applied for the measures with confirmed normality, and Spearman correlation was used for the other measures. The correlation type and coefficients are indicated on the plot. The correlation coefficients are given for the pooled age effect (left column under the correlation type) and separately by tone (right column, in the following order: Native /ga2/, Native /ga4/ and Non-Native /ga3/). The significance is illustrated by asterisks: **p<0.01, ***p<0.001, n.s. non-significant, Bonferroni-corrected across 14 measures and, when applicable, 3 tones.

**Supplementary Table 3:** LME analysis results for Early-latency Neural Encoding FFR vs Long-Latency Cortical LLR Measures

| Factor | DF1 | DF2 | F | p | η^2^_p_ |
| --- | --- | --- | --- | --- | --- |
| Age Category | 1 | 620 | 9.92 | 0.0017** | 0.02 |
| Tone | 2 | 620 | 0.51 | 0.5985 | 0.0016 |
| ERP type | 1 | 620 | 1.42 | 0.234 | 0.0023 |
| ERP measure | 2 | 620 | 0.12 | 0.8860 | 0.001 |
| Age Category × EPR type | 1 | 620 | 5.62 | 0.0174* | 0.01 |
| Age Category × Tone | 2 | 620 | 0.25 | 0.7800 | <0.001 |
| Tone × ERP type | 2 | 620 | 0.85 | 0.4267 | 0.0027 |
| Age Category × ERP measure | 2 | 620 | 0.73 | 0.4822 | 0.0023 |
| Tone × ERP measure | 4 | 620 | 0.08 | 0.9880 | 0.001 |
| ERP type × ERP measure | 2 | 620 | 0.82 | 0.4378 | 0.0026 |
| Age Category × Tone X ERP type | 2 | 620 | 0.64 | 0.5282 | 0.0021 |
| Age Category × Tone × ERP measure | 4 | 620 | 0.18 | 0.9490 | 0.0012 |
| Age Category × ERP type × ERP measure | 2 | 620 | 2.17 | 0.1156 | 0.007 |
| Tone × ERP type × ERP measure | 4 | 620 | 0.10 | 0.9821 | 0.001 |
| Age Category × Tone × ERP type × ERP measure | 4 | 620 | 0.25 | 0.9106 | 0.0016 |

**REFERENCES**

Anderson, S., Parbery-Clark, A., White-Schwoch, T., & Kraus, N. (2012). Aging Affects Neural Precision of Speech Encoding. *Journal of Neuroscience*, *32*(41), 14156–14164. https://doi.org/10.1523/JNEUROSCI.2176-12.2012

Anderson, S., Parbery-Clark, A., White-Schwoch, T., & Kraus, N. (2015). Development of subcortical speech representation in human infants. *The Journal of the Acoustical Society of America*, *137*(6), 3346–3355. https://doi.org/10.1121/1.4921032

Chang, C.-C., & Lin, C.-J. (2001). LIBSVM: a library for support vector machines. Retrieved from https://www.csie.ntu.edu.tw/~cjlin/libsvm/

Delorme, A., & Makeig, S. (2004). EEGLAB: An open source toolbox for analysis of single-trial EEG dynamics including independent component analysis. *Journal of Neuroscience Methods*, *134*(1), 9–21. https://doi.org/10.1016/j.jneumeth.2003.10.009

Eilers, P. H. C. (2003). A perfect smoother. *Analytical Chemistry*, *75*(14), 3631–3636. https://doi.org/10.1021/ac034173t

Gorga, M. P., Worthington, D. W., Reiland, J. K., Beauchaine, K. A., & Goldgar, D. E. (1985). Some Comparisons between Auditory Brain Stem Response Thresholds, Latencies, and the Pure-Tone Audiogram. *Ear and Hearing*, *6*(2), 105–112. https://doi.org/10.1097/00003446-198503000-00008

Hsu, C. W., & Lin, C. J. (2002). A comparison of methods for multiclass support vector machines. *IEEE Transactions on Neural Networks*, *13*(2), 415–425. https://doi.org/10.1109/72.991427

Jeng, F.-C., Peris, K. S., Hu, J., & Lin, C.-D. (2013). Evaluation of an Automated Procedure for Detecting Frequency-Following Responses in American and Chinese Neonates. *Perceptual and Motor Skills*, *116*(2), 456–465. https://doi.org/10.2466/24.10.PMS.116.2.456-465

Lakens, D. (2013). Calculating and reporting effect sizes to facilitate cumulative science: a practical primer for t-tests and ANOVAs. *Frontiers in Psychology*, *4*(NOV), 863. https://doi.org/10.3389/fpsyg.2013.00863

Lopez-Calderon, J., & Luck, S. J. (2014). ERPLAB: an open-source toolbox for the analysis of event-related potentials. *Frontiers in Human Neuroscience*, *8*(April), 1–14. https://doi.org/10.3389/fnhum.2014.00213

Parks, N. A., Gannon, M. A., Long, S. M., & Young, M. E. (2016). Bootstrap Signal-to-Noise Confidence Intervals: An Objective Method for Subject Exclusion and Quality Control in ERP Studies. *Frontiers in Human Neuroscience*, *10*(February), 50. https://doi.org/10.3389/fnhum.2016.00050

Skoe, E., & Kraus, N. (2010). Hearing It Again and Again: On-Line Subcortical Plasticity in Humans. *PLoS ONE*, *5*(10), e13645. https://doi.org/10.1371/journal.pone.0013645

Skoe, E., Krizman, J., Anderson, S., & Kraus, N. (2015). Stability and Plasticity of Auditory Brainstem Function Across the Lifespan. *Cerebral Cortex*, *25*(6), 1415–1426. https://doi.org/10.1093/cercor/bht311

Tallon-Baudry, C., Bertrand, O., Delpuech, C., & Pernier, J. (1996). Stimulus Specificity of Phase-Locked and Non-Phase-Locked 40 Hz Visual Responses in Human. *The Journal of Neuroscience*, *16*(13), 4240–4249.
